# *Cleonis pigra* (Scopoli, 1763) (Coleoptera: Curculionidae: Lixinae): Morphological Re-Description of the Immature Stages, Keys, Tribal Comparisons and Biology

**DOI:** 10.3390/insects10100325

**Published:** 2019-09-30

**Authors:** Jiří Skuhrovec, Semyon Volovnik, Rafał Gosik, Robert Stejskal, Filip Trnka

**Affiliations:** 1 Group Function of Invertebrate and Plant Biodiversity in Agro-Ecosystems, Crop Research Institute, Drnovská 507, CZ-161 06 Praha 6 Ruzyně, Czech Republic; 2 Independent Researcher, 72311 Melitopol, Ukraine; leucomigus@gmail.com; 3 Department of Zoology and Plant Protection, Maria Curie-Skłodowska University, Akademicka 19, 20-033 Lublin, Poland; cossonus@gmail.com; 4 Administration of Podyji National Park, Na Vyhlídce 5, CZ-669 02 Znojmo, Czech Republic; stejskal@nppodyji.cz; 5 Department of Ecology & Environmental Sciences, Faculty of Science, Palacký University Olomouc, Šlechtitelů 27, CZ-783 71 Olomouc, Czech Republic; filip.trnka88@gmail.com

**Keywords:** Coleoptera, Curculionidae, Lixinae, *Cleonis*, morphology, larva, pupa, biology, host plant, life history

## Abstract

Mature larvae and pupae of *Cleonis pigra* (Scopoli, 1763) (Curculionidae: Lixinae: Cleonini) are morphologically described in detail for the first time and compared with known larvae and pupae of other Cleonini species. The results of measurements and characteristics most typical for larvae and pupae of Cleonini are newly extracted and critically discussed, along with some records given previously. Keys for the determination of selected Cleonini species based on their larval and pupal characteristics are attached. Dyar’s law was used for the estimation of a number of larval instars of *C. pigra.* Descriptions of habitats, adult behavior, host plants, life cycle, and biotic interactions are reported here. Adults and larvae feed on plants from the Asteraceae family only (genera *Carduus*, *Cirsium*, *Centaurea*, and *Onopordum*). Oviposition occurs on the base of the plant stem or the root neck. In the process of larval development, a fusiform gall forms. *C. pigra* and *Cyphocleonus achates* can coexist in the same locality. In open habitats, the weevils become the prey of carnivorous animals.

## 1. Introduction

The tribe Cleonini belongs to the subfamily Lixinae [1], together with two other tribes: Lixini (approximately 40 genera, see [2]) and Rhinocyllini (two genera; sometimes part of Lixini, see [3]). Currently, Cleonini weevils include approximately 97 valid genus-group taxa [2,4] and 546 valid species [5,6,7]. The distribution is known to be mainly in the Northern Hemisphere; from south of the equator, they are known only in continental Africa and Madagascar [2]. Representatives of this tribe prefer xeric habitats and sandy soil. Their larvae are mono, oligo or polyphagous on herbs and shrubs, and they develop mainly in the lower parts of the host plant, especially the roots or, rarely, lower stems [2]. Endophagous larvae develop inside the plant tissue of the root neck or the collar of the host plants or create a characteristically swollen gall on the root of the host plants [8,9]. The morphology of immature Cleonini is still poorly known, but the first detailed, illustrated larval descriptions were recently made [9,10,11].

The members of the tribe Cleonini are known as a potentially significant pest of cultivated plants (beets, spinach, mayweeds, etc.), and they have the potential to be used as biocontrol agents against invasive Palaearctic plants [2]. For example, *Cyphocleonus achates* (Fåhraeus, 1842) has been used to control invasive spotted knapweed (*Centaurea stoebe* L., Asteraceae) in North America [12,13]. Another situation is known from Australia, where *Pachycerus segnis* (Germar, 1824) was tested for use against the invasive plant *Heliotropium europaeum* L. (Boraginaceae; as *P. cordiger*, [14]). Among the members of the tribe Cleonini, there are some other potential biological control agents, such as the weevil *Adosomus roridus* (Pallas, 1781), used against *Tanacetum vulgare* L., but this would be less effective and potentially dangerous due to its oligophagy [9]. Introducing and using such species as biological control agents might be risky for native fauna/flora, similar to the introduction of the weevil *Rhinocyllus conicus* (Froelich, 1792) [15].

The weevil *Cleonis pigra* (Scopoli, 1763) is a trans-Palaearctic species distributed from the Iberian Peninsula to the Far East [16]. It is also known from Central India [17]. The most northern areas of its range in Europe are southern Finland [18] and southern Norway (Ringerike, 60°12′19″ N; the Natural History Museum, University of Oslo). The most northern records of its appearance in Asia are approximately 60–63° N in Sakha (Yakutiya), Russia [19]. The beetle is common all over Ukraine [20] and in central and southern Europe, but is rather rare in the northern parts of the European distribution. However, this species was also registered in the Red List of Norway as vulnerable [21] and in the Red List of Finland [22]. In 1919, *C. pigra* was recorded in North America for the first time [23]. Recently, it has been recorded in a relatively small area in the northeastern USA and southeastern Canada, in the Atlantic region and nearby [23]. Accordingly, there is believed to be an adventive species in North America [23,24,25,26]. The immature stages of *C. pigra* were described by Cawthra [25] and Scherf [27], but setal nomenclature and morphological terms are not well understood, and some details in chaetotaxy and drawings are still missing.

Adult *C. pigra* were released in a testing pasture in Ontario, Canada, to decrease the population of *Cirsium arvense* (L.) Scop. [28], but further work has not been ongoing. The wide host range of *C. pigra* also includes the globe artichoke (*Cynara cardunculus scolymus* (L.) Hegi) [29,30], which is cultivated in many areas, including North America. Therefore, the beetle has not been considered a suitable biocontrol agent [31]. On the other hand, *C. pigra* is known as a pest of milk thistle (*Silybum* marianum (L.) Gaertn.), the seeds of which are important raw materials for pharmaceutics [32]. Incidentally, adults also feed on the sprouts of sunflowers (*Helianthus annuus* L. [33]) and non-Asteraceae plants; namely, Siberian pea shrub (*Caragana arborescens* Lam., Fabaceae; [34]), and beets (*Beta vulgaris* L., Amaranthaceae; see review [35]). Usually, all these damages occur only in spring and are insubstantial. Obviously, all data on the economic damage caused by *C. pigra* in beet plantations are based on misidentifications [35].

The main aims of this study are the following: (1) to re-describe larvae and pupae of *Cleonis pigra* in detail for the first time; (2) to compare this species with other known immature stages of this tribe; (3) to determine the number of larval instars via morphometric measurements; and (4) to provide details on their life history based on observations in central Europe.

## 2. Materials and Methods

### 2.1. Insect Collection and Laboratory Breeding

The material used to describe the larvae and the pupae was collected, and field observations were conducted in Ukraine in the following localities:

(1) Sheep pasture on the alluvial floodplain, near the Kamyana Mohyla Reserve (46°57′01″ N, 35°28′12″ E). Altitude: up to 11 m.a.s.l. Bedrock: sandy chernozem. Dominant plant species: *Cirsium ukranicum* Besser ex DC., and *Carduus hamulosus* Ehrh. (syn. *C. pseudocollinus* (Schmalh.) Klokov.), with occasional trees of *Elaeagnus commutata* Bernh. ex Rydb. growing among the grass (i.e., *Echinops ritro* subsp. *ruthenicus* L. (M. Bieb.) Nyman (syn. *E. ruthenicus*), and *Centaurea adpressa* Ledeb. ex Steud., *Melilotus albus* Medik.) in the clearings.
(2) Pishchanska Balka near Melitopol (46°49′50″ N, 35°20′18″ E). Altitude: up to 22 m.a.s.l. Bedrock: sandy chernozem with herbaceous covering. Dominant plant species: *Calamagrostis epigeios* (L.) Roth, *Linaria genistifolia* (L.) Mill., *Artemisia absinthium* L., *Echium vulgare* L., *Sisymbrium loeselii* L., *Achillea millefolium* L., *Hieracium umbellatum* L., *Melilotus albus* Medik., *Centaurea diffusa* Lam., *Cirsium arvense* (L.) Scop. (syn. *C. setosum* (Willd.) Besser), and *Chenopodium urbicum* L.

In the abovementioned localities, the life cycle, including feeding of adults and oviposition, was observed directly during the vegetation growing seasons of *Carduus hamulosus*, *Cirsium ukranicum*, and *Centaurea diffusa* from 2015 to 2017.

The author S.V. collected all larvae and pupae of *C. pigra* within roots of *Cirsium ukranicum*. Some stems and roots (*n =* 37) were dissected to investigate preimaginal development, and a further 150 plants were dissected to determine the quantity of preimaginal specimens of *C. pigra*. All photographs in the field were taken with a Canon PowerShot SX500 IS digital camera (Canon Inc., Ōta, Tokyo, Japan).

Laboratory observations were conducted in Melitopol, Ukraine (46°50′ N, 35°22′ E). The measurements of stems and roots were performed with a slide caliper and ocular micrometer. The size of root galls was determined at the greatest diameter. Adults were weighed on the Jadever electronic scale JKD-250 (Jadever Scale Co., Ltd, Taipei, Taiwan).

Geographical distribution and phenology were studied from several entomological collections, specifically, the Schmalhausen Institute of Zoology of National Academy of Sciences of Ukraine (Kyiv), the TG Shevchenko Kyiv the National University Zoological Museum, the Zoological Institute of the Russian Academy of Sciences (St. Petersburg), the VN Karazin Kharkiv National University Museum of Natural History, and Igor Maltsev’s (Odessa) and Sergey Suchkov’s (Melitopol) private collections. In total, more than 230 pinned specimens were studied. A virtual database of entomological collections of the Natural History Museum, University of Oslo (NHMUO) were used as well.

Unpublished data recorded in the previous project “Lixinae of Ukrainian steppe” (1981–1985) were also used here.

Adults of *C. pigra* were also collected close to the roots of *Cirsium eriophorum* (L.) Scop. in the central part of the Czech Republic in May 2014. The specimens were transported to the entomology laboratory at the Crop Research Institute (Prague, Czech Republic), and a breeding colony was established. Five male and female pairs were maintained in an insect chamber at 20 °C, with a relative humidity of 70% and a 12 h photoperiod. Females laid eggs, and after hatching, the young larvae were preserved in Pampel fixation liquid (see below).

### 2.2. Morphological Description

A part of each of the larval and pupal materials were preserved in Pampel fixation liquid (see [36]) and used for the morphological descriptions. The remaining specimens are deposited in the collection of the Group Function of Invertebrate and Plant Biodiversity in Agro-Ecosystems of the Crop Research Institute (Prague, Czech Republic). The insect host plant was identified by a plant taxonomist. Insect slides were prepared according to the May [37] guidelines.

The observations and measurements were conducted using a light microscope with calibrated oculars (Olympus BX 40, SZ 11, both Olympus, Shinjuku, Tokio Prefecture, Japan; and Nikon Eclipse 80i, Nikon, Minato, Tokio Prefecture, Japan). The following characteristics were measured for each larva: head width, length of the body (larvae fixed in a C-shape were measured in the middle of the segments in lateral view), and width of the body in the widest place (i.e., meso- and metathorax). For the pupae, the length and the width at the widest place were measured. The lengths of all setae are visible in the figures.

Drawings were made with a drawing tablet (Intuos Pro S, Wacom, Saitama Prefecture, Japan), and the digital images were subsequently processed with Adobe Photoshop (Adobe Inc., San Jose, CA, USA), Corel Photo-Paint 11 (Corel, Ottawa, ON, Canada), and/or GIMP 2 (GIMP Development Team, Charlotte, NC, USA). The numbers of setae of bilateral structures are given for one side.

We used the terms and abbreviations for the setae of the mature larvae and pupae according to Scherf [27], May [37,38], and Marvaldi [39,40].

All morphological abbreviations used in text: 

**Abd. I–X**—abdominal segments 1–10, **Th. I–III**—thoracic segments 1–3, **at**—antenna, **st**—stemmata, **lr**—labral rods, **ur**—urogomphi; setae: ***als***—anterolateral, ***ams***—anteromedial, ***as***—alar (larva), ***cls***—clypeal, ***d***—dorsal (pupal abdomen), ***des***—dorsal (larval head), ***dms***—dorsal malar, ***ds***—discal (pupal prothorax), ***ds***—dorsal (larval abdomen), ***eps***—epipleural, ***eus***—eusternal, ***fes***—femoral, ***fs***—frontal, ***les***—lateral epicranial, ***ligs***—ligular, ***lms***—labral, ***l***, ***ls***—lateral, ***lsts***—laterosternal, ***mbs***—malar basiventral, ***mds***—mandibular, ***mes***—median, ***mpxs***—maxillary palp, ***os***—orbital, ***pas***—postantennal, ***pda***—pedal, ***pds***—postdorsal, ***pls***—posterolateral, ***pes***—postepicranial, ***pfs***—palpiferal, ***pms***—postmental, ***prms***—premental, ***prns***—pronotal, ***prs***—prodorsal, ***ps***—pleural, ***rs***—rostral, ***sls***—super lateral, ***sos***—super orbital, ***ss***—spiracular, ***stps***—stipal, ***sts***—sternal, ***trs***—trochanter, ***ts***—terminal, ***v***—ventral (pupa), ***ves***—ventral epicranial, ***vms***—ventral malar, ***vs***—vertical.

## 3. Results and Discussion

### 3.1. The Morphology of Immature Stages of Cleonis pigra

#### 3.1.1. Materials Examined

Larvae: Ukraine, Zaporizhia Province, near the Kamyana Mohyla Reserve (46°57′01″ N, 35°28′12″ E). Three mature larvae, collected on 12 August 2015 in the galls on the root necks of *Cirsium ukranicum*; Slovakia, Brestovany: one mature larva, 21 July 2012.

Pupae: Ukraine, Zaporizhia Province, near the Kamyana Mohyla Reserve (46°57′01″ N, 35°28′12″ E). Four ♂♂ and three ♀♀, collected on 12 August 2015 in the galls on the root necks of *Cirsium ukranicum*.

#### 3.1.2. Description of Mature Larvae

Measurements (in mm, *n =* 4). Body length: 14.8–17.2. Body width: (metathorax and abdominal segment I) 4.40–5.33. Head width: 2.45–2.80.

**Figure 1:**
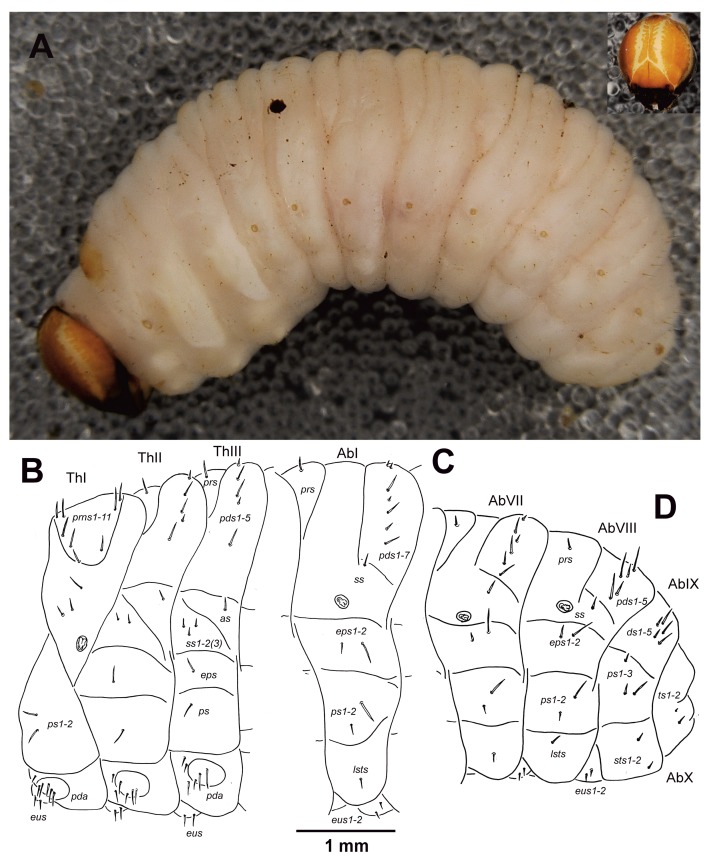
*Cleonis pigra* mature larva, habitus. (**A**)—shape of the body (lateral view); (**B**)—lateral view of thoracic segments; (**C**)—lateral view of abdominal segment II; (**D**)—lateral view of abdominal segments VII–X. (*prns*—pronotal seta(e), *prs*—prodorsal s., *pds*—postdorsal s., *as*—alar s., *ss*—spiracular s., *eps*—epipleural s., *ps*—pleural s., *pda*—pedal s., *lsts*—laterosternal s., *eus*—eusternal s., *ds*—dorsal s., *sts*—sternal s., *ts*—terminal s.; Th1–3 and Ab1–10—number of thoracic and abdominal segments).

General. Body stocky, slightly curved, rounded in the cross section (Figure 1A). Cuticle without any spiculation.

**Figure 2:**
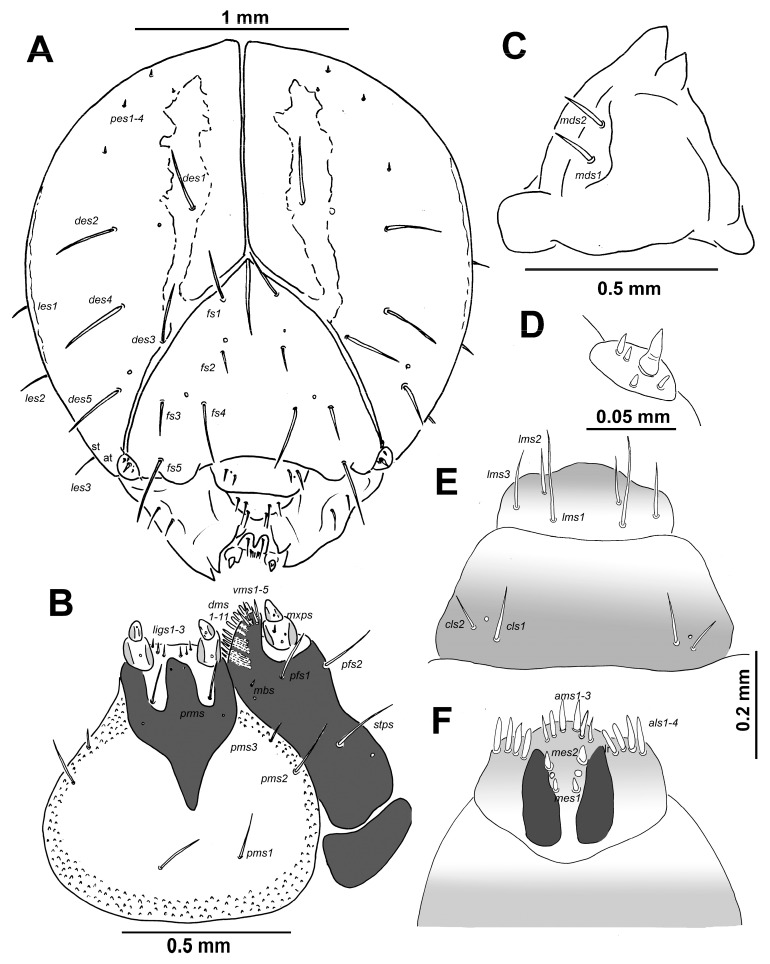
*Cleonis pigra* mature larva, head, antenna, and mouth parts. (**A**)—dorsal view (*des*—dorsal epicranial seta (e), *fs*—frontal epicranial s., *les*—lateral epicranial s., *ves*—ventral epicranial s., st—stemmata, at—antenna); (**B**)—right maxilla, dorsal view (*dms*—dorsal malar s., *vms*—ventral malar s., *mpxs*—maxillary palps s., *mbs*—basoventral s., *pfs*—palpiferal s., *stps*—stipital s.), prementum and postmentum, ventral view (*prms*—premental s., *pms*—postmental s., *ligs*—ligular s.); (**C**)—right mandible (*mds*—mandible dorsal s.); (**D**)—antenna; (**E**)—labrum and clypeus (*cls*—clypeal s., *lms*—labral s.); (**F**)—epipharynx (*ams*—anteromedial s., *als*—anterolateral s., *mes*—median s., lr—labral rods).

Coloration. Light brown or brown head with a distinct pale pattern around the frontal line (Figure 1A). All thoracic and abdominal segments white; only dorsum of pronotum with elongated light brown stripe (Figure 1A and Figure 2A).

Vestiture. Setae thin; short to long; orange (Figure 1A).

Head capsule (Figure 2A). Head suboval, slightly longer than wide, endocranial line weak, and as long as a one-third the length of frons. Frontal sutures on head distinct, but narrow; Y-shaped and extended to stemmata. Single stemma (st), in the form of a dark pigmented spot, located on each side anterolaterally. *Des_1_* and *des_2_* located in upper part of the central part of epicranium, *des_1_* near to the middle part of epicranium, and *des_2_* near to side of epicranium, *des_3_* located anterially near to frontal suture, *des_4_* located in the central part of epicranium, *des_5_* located anterolaterally; all *des* very long, and almost all equal in length (Figure 2A). *Fs_1_* and *fs_2_* placed medially, *fs_3_* located anteriomedially, *fs_4_* located anteriolaterally, and *fs_5_* located laterally, close to the epistoma; *fs_1_*, *fs_3_* and *fs_5_* very long; *fs_2_* and *fs_4_* relatively long to long, as long as half-length of the remaining three setae (Figure 2A). *Les_1–3_* and *ves_1_* as long as all *des*; and *ves_2_* relatively long. Epicranial area with four *pes* in line with upper *des_2_*.

Antennae located at the end of the frontal suture on each side; membranous and convex basal article bearing conical triangular sensorium, very long; basal membranous article with four sensillae different in both shape and length (Figure 2D).

Clypeus (Figure 2E) is approximately 2.2 times as wide as long with two relatively long to long *cls*; *cls_2_* distinctly shorter than *cls_1_*, localized posteriolaterally. One sensillum; anterior margin rounded to the inside; median part covered by thorn-shaped cuticular processes.

Mouth parts. Labrum (Figure 2E) approximately 2.5 times as wide as long, with three piliform *lms*, of different length; *lms_2_* and *lms_3_* distinctly shorter than very long *lms_1_*; *lms_1_* is placed close to the margin with clypeus, *lms_2_* is located anteriomedially, and *lms_3_* is located anteriolaterally; anterior margin double sinuate. Epipharynx (Figure 2F) with four blunt, finger-like *als*, unequal in length; two laterally *als* distinctly longer than two medially *als*; three *ams*, *ams_1_*, and *ams_2_* blunt, finger-like, distinctly larger than piliform *ams_3_*; two very short to short, blunt *mes*, unequal in length, both located close to lr; and one sensillum close to *mes_2_*; labral rods (lr) elongated, triangularly, converging anterially. Mandibles (Figure 2C) distinctly broad, bifid, tooth of unequal height; slightly truncate; both *mds* relatively long, hairform, located in distinct holes. Maxilla (Figure 2B) stipes with one *stps*, two *pfs* and one *mbs*; *stps* and *pfs_1-2_* very long, equal in length; *mbs* very short. Mala with 11 *dms* in two different lengths (six bacilliform relatively long and five piliform long to very long); five *vms*, four as long as bacilliform *dms*, and one very short. Maxillary palpi with two palpomeres: basal palpomere with one very short *mxps* and two sensilla; length ratio of basal and distal palpomeres: 1:0.6; distal palpomere with one sensillum and a group of conical, cuticular apical processes. Praelabium (Figure 2B) heart-shaped and partially elongated, with one very long *prms*; ligula with sinuate margin and three hairform short *ligs*, unequal in length; premental sclerite, ψ-shaped, and well sclerotized. Labial palpi with two palpomeres; length ratio of basal and distal palpomeres: 1:0.5; distal palpomere with one sensillum and short, cuticular apical processes; basal palpomere with one dorsal sensillum. Postlabium (Figure 2B) with three *pms*: *pms_1_* located anterially, remaining two pairs laterally; *pms_1_* and *pms_2_* very long; *pms_3_* as long as one third-length of the previous two setae. Surface of postlabium partly covered by distinct cuticular processes.

Thorax. Prothorax distinctly smaller than meso and metathorax. Spiracle bicameral. Prothorax (Figure 1B) with 11 *prns* unequal in length, eight of them on weakly pigmented dorsal sclerite; this sclerite is subdivided into two triangular plates medially, three of setae closely to the spiracle; two relatively long *ps* and two relatively long *eus*. Mesothorax (Figure 1B) with one *prs*, five *pds* unequal in length; *pds_1_* short to relatively long, *pds_2_*_–*3*_ and *pds_5_* relatively long to long, *pds_4_* short; one short to relatively long *as*; two *ss* equal in length, both short; one relatively long *eps*; one relatively long *ps* and two relatively long *eus*. Chaetotaxy of metathorax (Figure 1B) almost identical to mesothoracal, metathorax with two or three *ss* almost equal in length, all short. Each pedal area of thoracic segments well separated, with six relatively long to long *pda*, and one short *pda*.

Abdomen. Abdominal segments I–IV are of almost equal length, with subsequent abdominal segments decreasing gradually to the terminal parts of the body. Abdominal segment X reduced to four anal lobes of unequal size, the dorsal being distinctly the largest, the lateral pair equal in size, and the ventral lobe very small. Anus located terminally. Spiracles bicameral, the eight abdominal spiracles located laterally, close to the anterior margin of abdominal segments I–VIII. Abdominal segments I–VII (Figure 1C) with one short *prs*; seven *pds* unequal in length, *pds_1_*_–*2*_, *pds_4_*, and *pds_6_* short to very short, *pds_3_*, *pds_5_* and *pds_7_* long, but abdominal segment VII without *pds_1_*; one short to relatively long *ss*; two *eps* of unequal length, *eps_1_* short, *eps_2_* long; two *ps* of unequal length, *ps_1_* short, *ps_2_* long; one short *lsts* and two short *eus*. Abdominal segment VIII (Figure 1D) with one short *prs*; five *pds* unequal in length, *pds_4_*,*_6_* short to very short, *pds_3_*,*_5_*,*_7_* long, *pds_1_*_–*2*_ absent; one short to relatively long *ss*; two *eps* of unequal length, *eps_1_* short, *eps_2_* long; two *ps* of unequal length, *ps_1_* short, *ps_2_* long; one short *lsts* and two short *eus*. Abdominal segment IX (Figure 1D) with five *ds* (*ds_1_*_,*3*,*5*_ long and *ds_2_*_,*4*_ short); three *ps* of unequal length, *ps_1_* very short to minute, but *ps_2_*_–*3*_ almost as long as *ds_1_*; and two very short to minute *sts*. Lateral anal lobe on abdominal segment X (Figure 1D) with two very short to minute setae (*ts*).

#### 3.1.3. Description of Pupae

Measurements (in mm; four ♂♂, three ♀♀). Body lengths: ♂ 13.7–15.6 (mean 14.9) and ♀ 14.8–16.7 (mean 16.5). Body widths: ♂ 5.1–5.6 (mean 5.5) and ♀ 6.0–7.5 (mean 6.7). Thorax widths: ♂ 4.7–4.9 (mean 4.5) and ♀ 3.8–4.5 (mean 4.5). Head widths: ♂ 2.2–2.3 (mean 2.2) and ♀ 2.3–2.4 (mean 2.3).

Coloration. All thoracic and abdominal segments light yellowish. Cuticle smooth, except thorn-like processes on abdominal segments II–VIII.

**Figure 3:**
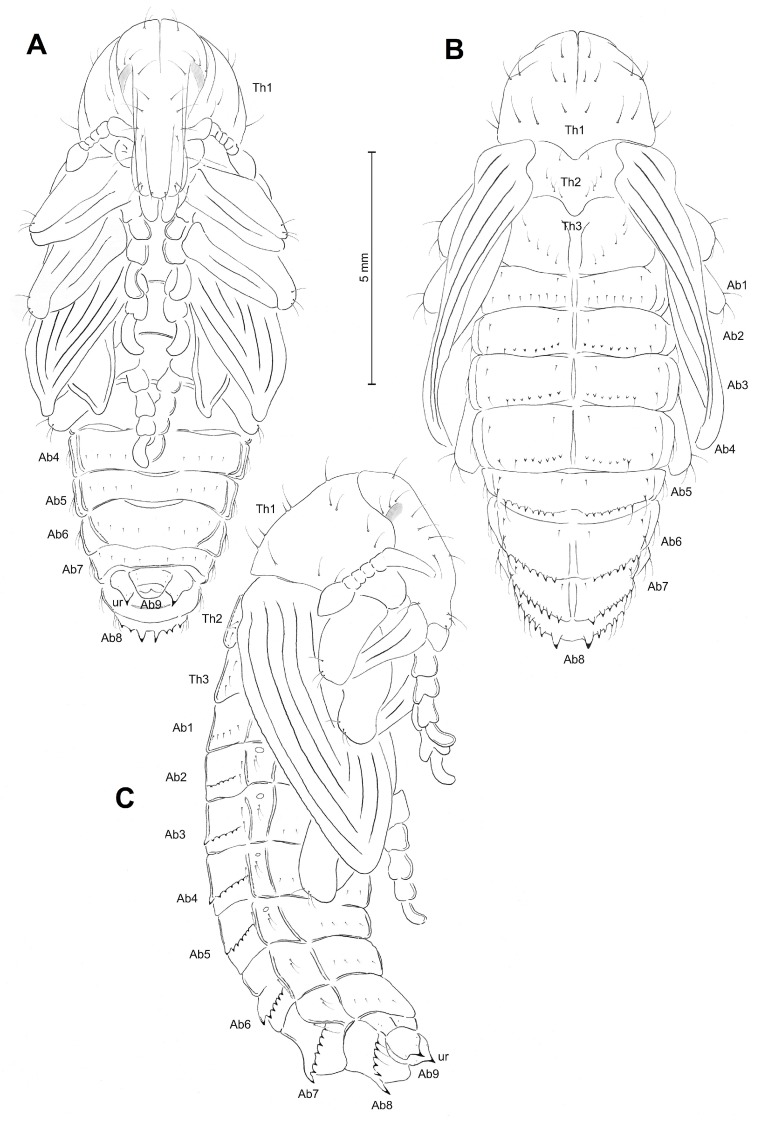
*Cleonis pigra* pupa, habitus. (**A**)—dorsal view; (**B**)—ventral view; (**C**)—lateral view (Ab1–9—number of abdominal segments, Th1–3—number of thoracic segments, ur—urogomphi).

**Figure 4:**
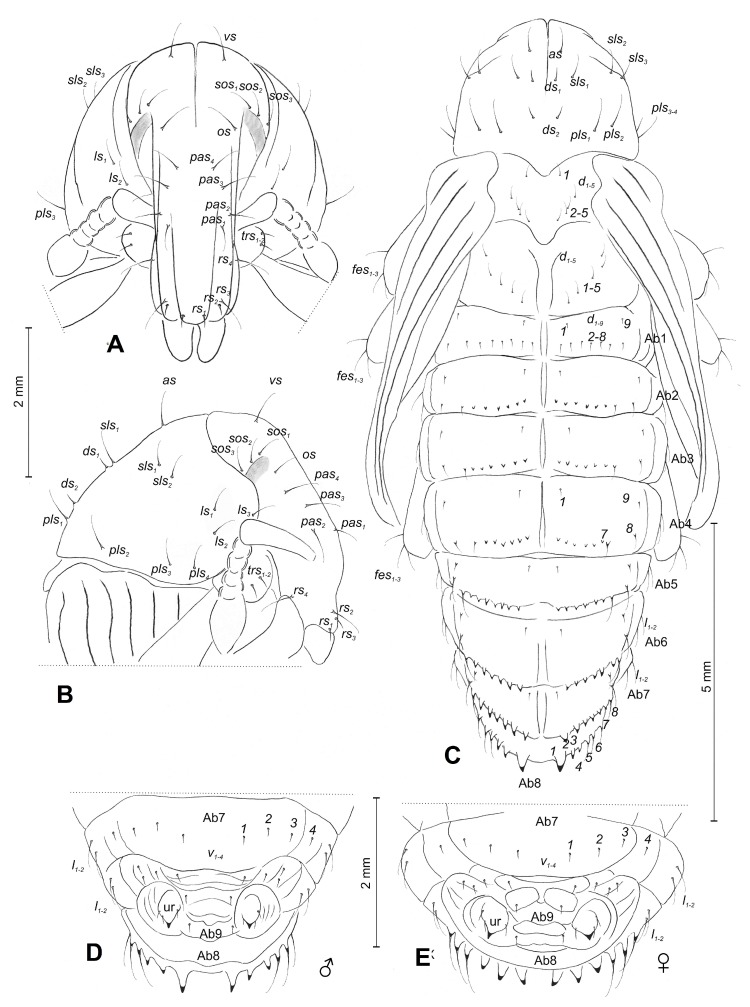
*Cleonis pigra* pupa, chaetotaxy. (**A**)—head and rostrum; (**B**)—lateral view of head and pronotum; (**C**)—dorsal view; (**D**)—ventral view of last abdominal segments of male; (**E**)—ventral view of last abdominal segments of female (Ab1–9—number of abdominal segments, Th1–3—number of thoracic segments, ur—urogomphies. Setae: *l*, *ls*—lateral, *sls*—super lateral, *d*—dorsal, *ds*—discal, *pls*—posterolateral, *trs*—trochanters, *v*—ventral, *vs*—vertical, *sos*—super orbital, *os*—orbital, *pas*—postantennal, *rs*—rostral, *fes*—femoral).

Morphology (Figure 3A–C). Body slender and elongated. Rostrum long, about three times as long as wide, and extended to mesocoxae. Antennae rather short. Pronotum 1.4 times as wide as long. Mesonotum slightly shorter than metanotum. Abdominal segment I rather short; abdominal segments II–IV of equal length, longer than abdominal segment I; abdominal segment VI relatively long; abdominal segment VII almost semicircular; abdominal segments VIII and IX distinctly smaller than other segments. Urogomphi rather short, conical, with sclerotized apexes. Spiracles placed laterally; on abdominal segments I–V functional, and on abdominal segment VI atrophied; on next segments spiracles invisible. Sexual dimorphism visible in the structure of abdominal segment IX: gonotheca of ♂ undivided (Figure 4D), ♀ divided (Figure 4E).

Chaetotaxy (Figure 4A–E). Setae distinct, with different lengths; hair-like; light yellow. Head capsule includes one *vs*; three *sos* equal in length; and one *os* and four *pas* equal in length. Rostrum with four *rs*, different in length: *rs_1_* and *rs_2_* very short and *rs_3_* and *rs_4_* long; *rs_1_*_–*3*_ located apically and *rs_4_* latero-apically.

Pronotum with one *as*, three *sls*, two *ds*, four *pls*, and three *ls*; discal setae very short, remaining setae elongated, equal in length (Figure 4B). Mesonotum with five setae (*d*) of which: the first is located antero-medially; the next four form a diagonal line medially. Metanotum with five setae (*d*) forms a diagonal line medially. All setae on meso and metanotum are equal in length (Figure 4C). Trochanter of prolegs with two trochanters setae (*trs_1–2_*) (Figure 4A). Each apex of femora with three elongated *fes* (Figure 4C).

Abdominal segment I with nine short, hair-like setae of which: *d_1_* is located antero-medially, *d_2–8_* are along posterior margin of segment, *d_9_* is located anterolaterally. Setae *d_2_*_–*7*_ on abdominal segments II–VII replaced by thorn-like cuticular processes. Setae *d_7_* and *d_8_* are alike; cuticular processes increase from abdominal segments II to VII, while *d_1_* and *d_9_* stay as short as those on abdominal segment I. Abdominal segment VIII has one very short antero-medially seta, three thorn-like cuticular processes (of which first distinctly longer than next), all distributed along posterior margin, and finally, three elongated postero-laterally setae placed on protuberances. Each of lateral parts of abdominal, segments I–VIII, have one very long seta. Each ventral part of abdominal segments I–VIII has four short setae, forming a regular, horizontal line on median parts. Abdominal segment IX has three short setae placed on urogomphi and next single setae on gonothecae.

#### 3.1.4. Remarks on the Previous Descriptions of Immature Stages of *Cleonis pigra*

The larval description of *C. pigra* by Scherf [27] used a different setal nomenclature and morphological terms, rendering understanding difficult. The chaetotaxy of the head described by Scherf [27] is questionable because he listed only four *fs*, three or four *vms*, and 12 *dms*. In contrast, we listed the presence of five *fs*, five *vms*, and 11 *dms* (Figure 2A,B). Scherf [27] also listed that the labrum and epipharynx have nine setae, but we observed three *lrms*, three *ams*, four *als*, and two *mes*. Some of the setae on the epipharynx (especially *ams* and *mes*) cannot be compared exactly due to unclear drawings and unclear resolution of the distal *mes*, which some authors listed as *ams* (see [41]). The chaetotaxy of the body is more problematic due to missing drawings, and the count of setae is questionable (e.g., [27] listed 23 setae on the pronotum, but we observed only 22 setae). Cawthra [25] presented some drawings of the mature larva of *C. pigra*, but understanding them is problematic.

The pupal description by Scherf [27] is confusing and different from our observations, he listed only one *rs*, two *fes*, eight setae on the pronotum, and six setae on the mesonotum. In contrast, we listed the presence of four *rs*, three *fes*, 13 setae on the pronotum, and five setae on the mesonotum (Figure 4C).

#### 3.1.5. A Comparison with Larvae of Other Cleonini

The larvae of twelve Cleonine taxa have already been described in previous studies [9,10,11,27,42,43], and a detailed description of the pupae is known for only eleven Cleonine taxa [9,10,11,27,43]. The comparison of the larva and pupa of *Cleonis pigra* with those described by Hoffmann [42] and Scherf [27] was somewhat difficult due to the use of different terminology for morphology and chaetotaxy and/or the absence of good quality drawings (see [9]). Despite these challenges, we were able to compare the morphology of eleven known taxa (except description from [42]) and give tha comparison in the Key (see Key for the known immature stages of Cleonini).

The mature larvae of the subfamily Lixinae were characterized by three diagnostic features [44]: the increased number of *pds* (1) on the meso- and metathorax and (2) abdominal segments I–VII, and (3) the increased number of setae on the epipharyngeal lining (*als*); i.e., more than the most frequent number of setae in weevils (for details, see [11]). These differential features of mature larvae were confirmed in all known descriptions from the tribe Cleonini [9,10,11,27,43], and all known species from the Lixini tribe fit this diagnosis (genus *Larinus* [45,46,47,48]; *Lixus* species [27,45,49,50,51,52,53,54,55]; and *Rhinocyllus conicus* [37]).

The larval morphological features, such as (1) the presence of endocarina (Figure 2A), (2) the count and position of setae on the labrum (Figure 2E), and (3) the count of setae on the mandible (Figure 2C), seem to be important features for generic study of the Cleonini. The key for all known immatures from the tribe Cleoinini is presented below (see Table 1). The detailed generic study of the Cleonini tribe and the comparison of both tribes is not still possible because of our limited knowledge of the immature stages. However, all these data confirm that the detailed descriptions of immature stages are very important for further studies on generic and suspected generic taxonomic relationships within Lixinae, as well as for the effective protection of endangered species, the exploitation of their potential in life science, and the promotion of using larvae of Cleonini species as potential biological control agents against weeds (e.g., *Carduus*, *Cirsium*, *Tanacetum*) [9]. Although the number remains low in comparison with the total number of Cleonini species, these results demonstrate the possibility of identifying the immature stages in these species, as was done in other groups of weevils (see *Otiorhynchus* [56], Tychiini [57,58,59], and Mecinini [60]).

#### 3.1.6. Key to the Known Immature Stages of Cleonini

##### Larvae (Last Instar)

The key is based on recently detailed descriptions of mature larvae of *Cleonis pigra* and ten descriptions of larvae published before [9,10,11,27,43] (Table 1).

##### Pupae

The key is based on recent descriptions of pupa of *Cleonis pigra* and descriptions of pupae published before [9,10,11,43] (Table 2).

#### 3.1.7. Measurements of Larval Instars

Cawthra [25] and Scherf [27] concluded that larvae of *C. pigra* have four instars. We calculated the hypothetical cephalic width according to Dyar’s law (the observed model that increases in sclerotized body parts during development of the arthropod’s immature stages are predictable and normal by a relatively constant factor) using the ratios 1.35, 1.40, and 1.50 [61,62] because we were able to measure the head capsule only for the first instar and the last instar. The best approximation to the real size was obtained for four instars as in Scherf [27], but we had small discrepancies in the measured size in the first and fourth instars. Cawthra [25] listed the following ranges of head width of each instar: L1 0.77–0.92 mm; L2 1.08–1.31 mm, L3 1.54–1.93 mm, and L4 2.16–2.62 mm. Scherf [27] listed identical measurements as Cawthra [25], but he did not mention this paper. On the other hand, we measured a small head width for both instars: L1 0.99–1.17 mm and L4 2.45–2.80 mm.

### 3.2. Biology and Ecology of Cleonis pigra

#### 3.2.1. Habitats

**Figure 5:**
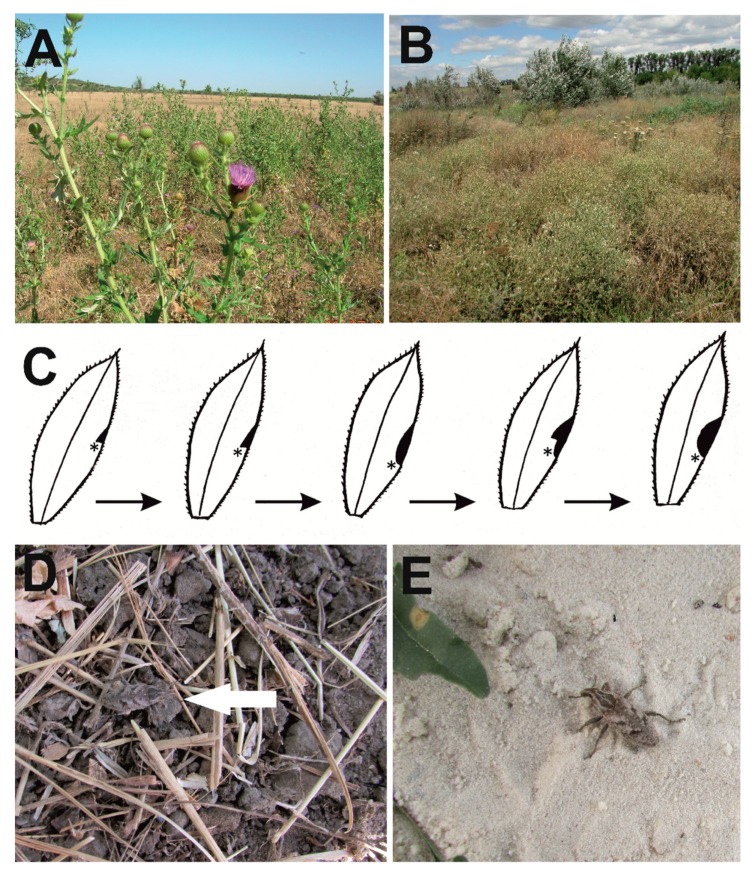
*Cleonis pigra* habitats. (**A**)—habitat with flowering *Cirsium ukranicum* (near the Kamyana Mohyla Reserve); (**B**)—habitat with flowering *Centaurea diffusa* (Pishchanska Balka); (**C**)—the schema how adult of *Cleonis pigra* eats a leaf of *Cirsium setosum*. Asterisks mark the beetle location; (**D**)—*Cleonis pigra* on the roadside; (**E**)—*Cleonis pigra* on the seashore. All photos: S. Volovnik.

The weevil *Cleonis pigra* occurs predominantly in coastal biotopes, forest edges and glades, man-made treelines, roadsides, quarries, wastelands and other ruderal plots, and pastures (Figure 5A,B). Adults are usually recorded on the soil surface or in the upper soil level. Sometimes, they are locally rather numerous. They can be found under stones [63]. This weevil prefers biotopes with light sandy soils. In the southwest of Hungary, it inhabits the recultivated dumps of uranium mines [64]. In the Czech Republic, *C. pigra* prefers disturbed habitats, such as roadsides, fallow land, and waste places [65]. In Turkey, an adult was recorded as high as 2450 m.a.s.l. in the mountains [66].

Quite often, *C. pigra* is encountered in agricultural lands-fields [67], plantations, orchards, and vineyards [68]. In western Ukraine, *C. pigra* accounted for more than half of the total weevils recorded in sugar beet plantations [69]. Adults are attracted there by an abundance of Asteraceae weeds. As a result, numerous beetles of the new generation are found in combined oats [70,71] and in bagged and stored beans [70,72] during or after harvesting.

#### 3.2.2. Adult Behavior

The weevil *Cleonis pigra* is active in the day. In April, motionless adults are often found on the places warmed by the sun. They feed on the leaves and are most active in warm dry weather. Usually, a feeding adult can be found on the lower surface of the leaf, near its margin. Without moving, the adult bites and rips pieces of mesophyll tissue of leaves and young sprouts. When the apex of its rostrum reaches the most distant point, the weevil begins to gnaw from the starting point again. This results in semicircular apertures in the leaves (Figure 5C). If the leaf is large, the inner edges of apertures do not reach the midrib. The adult did not eat the small spines along the margins of the leaf (*Cirsium*, *Carduus*, and *Onopordum*). These spines fell down when the weevil ate the soft tissue around their base. Occasionally, a beetle creates this round hole in a leaf blade (*Arctium*). *Cleonis pigra* is capable of fasting for an average of 28 days [73]. When adults do not feed, they are found on the underside or on the base of leaves or near the base of the main stem (*Cirsium*, *Carduus*).

In response to a slight disturbance, *C. pigra* beetles climb on the underside of the leaf they are currently on or fall to the ground and go into thanatosis. One of the common names of *C. pigra* is “sluggish weevil”. The color pattern of its covering makes it barely visible on the grey soil and plant debris, but does not hide it on the coastal sands (Figure 5D,E). Therefore, these beetles often become victims of water birds (see below—Biotic interactions). To move from the upper side of the leaf to the lower side, the beetle goes to the leaf margin, moves its forelegs to the lower side and turns to the lower side in one sharp heave. *C. pigra* beetles have long wings and unfused elytra, but we have never seen them flying. Sometimes, an abruptly caught beetle releases a drop of semi-liquid greenish excrement.

#### 3.2.3. Host Plant

In Ukraine, we recorded both adults and larvae feeding on *Cirsium ukranicum* Besser ex DC., *C. arvense* (L.) Scop., *Carduus nutans* L., *C. hamulosus* Ehrh., *C. uncinatus* M. Bieb., *Centaurea diffusa*, *C. odessana* Prodan, and *Onopordum acanthium* L. Imago fed on *Arctium* L., *Taraxacum* F. H. Wigg., and *Hieracium* L. [35]. Isaev [74] reared imago from the root of *Jurinea cyanoides* (L.) Rchb. In the Czech Republic and Slovakia, frequent hosts of *Cleonis pigra* are *Cirsium vulgare* (Savi) Ten., *Carduus acanthoides* L., and *Arctium* species. Less often, the larvae were also found in *Centaurea stoebe* in southern Moravia (R. Stejskal, pers. observ.).

According to Batra et al. [75], in the field, *Cnicus* L. and *Silybum* Vaill. were also recorded as host plants for *C. pigra*. In laboratory tests, beetles fed on the plants of 17 genera, exclusively Asteraceae [75]. Thus, it is oligophagous (first-degree oligophagy *sensu* [76]). In the field, imago prefers larger plants [77].

In northern Ukraine, over 90% of populations of *Cirsium arvense* and approximately 40% of the population of *Onopordum acanthium* were attacked by larvae of *C. pigra* [69]. In European populations, only 7% of *Carduus nutans* plants were attacked by *C. pigra* [78], but up to 18% [79] or even 80% [25] of *Cirsium arvense* plants were attacked by *C. pigra*. In Canada, beetles were recorded on thistle (*Cirsium arvense*) 12% of the time, on average [80]. High host plant density results in a high number and density of herbivores. The average density of *C. pigra* in the monoculture of *Silybum marianum* reaches almost 21 larvae on 25 plants [32]. Throughout spring to the end of summer, infection of plants by *C. pigra* significantly decreases because over 30% of immature instars die [25]. However, in small plots (up to 0.01 ha), the plant rate infestation, at times, may reach 100% [81].

If *Cirsium arvense* and *Carduus tenuiflorus* Curt. grow at the same locality, *C. pіgra* prefers only the first [25]. Generally, it seems that *Cirsium* is the most preferable host plant [73]. According to Anderson [23], this weevil has no host races. Interestingly, there is no information about *C. pigra* on *C. diffusa* in North America where both species are adventive.

#### 3.2.4. Life Cycle

In Ukraine, adults were recorded from late April (Eupatoria, Yevpatoria, East Crimea) onwards. In the Czech Republic, the first adults are found earlier, in March [65]. In the northeastern USA, adults appear later, in May [82]. Active imagines from the new generation were observed from the second part of August to the end of October (Podilsk, Odessa Province). Once, an active adult was found as late as the beginning of December [83].

In the spring, the adults feed first on the rosettes of the host plants and then gnaw the young leaves on the shoots. Mating and oviposition occur from the end of April to the end of June. In the laboratory, males and females mated more than once and with different partners. A mating couple was observed by one author (S.V.) in copula for one hour.

Females prefer to oviposit on plants with a larger root crown diameter and a higher number of shoots [84]. *Cirsium arvense* is dioecious, and according to Jung (cited by [79]), the roots of its female plants are attacked by *Cleonis pigra* three times more frequently than the roots of its male plants. A female of *C. pigra* searches for a suitable site for egg laying on the base of the host plant stem or root neck. Usually, this point is located partly under the soil surface. The female then makes a short tunnel through the soil with her rostrum, gnaws the pit into the cortex, and oviposits. Freshly laid eggs are matted, yellowish, light oval, 1.4–1.8 mm long, and 1.1–1.3 mm wide. The eggs are laid solitarily. The period of oviposition lasts up to the end of June [83]. Detailed descriptions of egg-laying behavior are provided in Cawthra [25].

**Figure 6:**
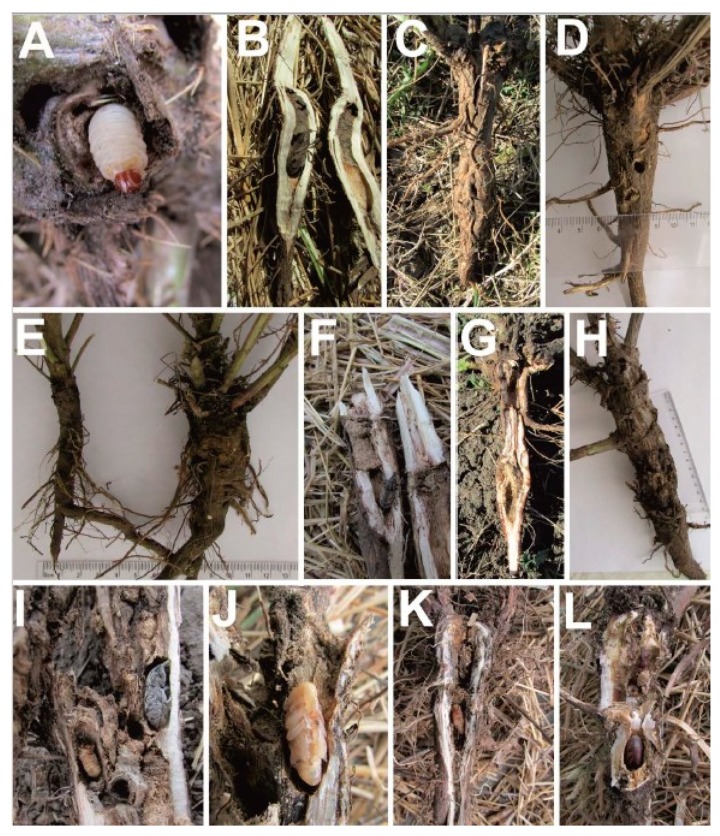
*Cleonis pigra* life history. (**A**)—mature larva in the root crown of *Cirsium ukranicum*; (**B**)—the curved tunnel, pupa chamber and adult; (**C**)—root gall of *C. ukranicum*; (**D**)—normal root of *Centaurea diffusa* (left) and gall on the same plant; (**E**)—root gall on *C. ukranicum*; (**F**)—adult in the larval chamber with cork above the chamber; (**G**)—larval tunnel and pupal chamber on *C. ukranicum*; (**H**)—a compound gall on *C. ukranicum*; (**I**)—adult and dead larva in a gall; (**J**)—young pupa of *Cleonis pigra*; (**K**)—pupa begins to sclerotize from its eyes and rostrum; (**L**)—occasionally, pupa and adult may be located head down. All photos: S. Volovnik.

In the laboratory, larvae hatched after 6–9 days (10–12 days according to [35]). In the field, young larvae were found from the beginning of April to mid-June. Larvae attacked the central part of the root where the vascular tissue is located. The mature larva coloration was milky white (Figure 6A). Larvae gnawed tunnels downward, up to 4–5 mm in diameter. The tunnels may be straight or slightly curved (if this part of the root is curved as well) (Figure 6B). All observed by one author (S.V.).

In the process of larval development, a fusiform gall was formed on the root collar (*Carduus hamulosus*, *Cirsium ukranicum*) or on the taproot (*C. hamulosus*, *C. ukranicum*, *Centaurea diffusa*, and *Centaurea odessana*) [8] (Figure 6C–E). The weevil also formed galls on *Centaurea stoebe* (syn. *C. maculosa* Lam.) [85]. The beginning of gall formation became visible at the end of the first instar of larval development. Root galls were typically located at a depth of 10–30 mm below ground level, although some were found up to 60 mm below ground level. As a rule, galls were located on the main root. Due to gall formation, growing larva stops moving and feeds by growing tissues around itself. This may result in the formation of a cavity with some dust (e.g., bits of vascular and other plant tissues).

The larval stage lasts approximately 30 days [82]. Finishing its development, a larva gnaws a pupa chamber from 15–50 mm (in *Centaurea odessana* and *Cirsium ukranicum*, respectively) below the base of the root. The length of the chamber is 12–19 mm and the width is 6–7 mm (i.e., the chambers are slightly wider than the larval tunnel). This chamber may be situated below, above, or adjacent to the cavity of the tunnel. Before or after the construction of the chamber, larvae compacted bits of plant tissue and excrement as a type of cork above or below the chamber (Figure 6F). This “cork” may be up to 10 mm in length. In another case, larvae compacted the dust on the walls of the chamber (Figure 6G).

Occasionally, several larvae were found mining in the same plant specimen. Therefore, their galls were formed one above another or/and side by side. A plant usually has one or two (and less frequently, up to six) galls. Typically, adjacent galls merge as they grow (but the larval tunnels and pupal chambers remain isolated). One such compound gall on *C. ukranicum* reached a size of 55 × 110 mm, whereas the diameter of the base of the stem was 26 mm (Figure 6H). In the spring, we found up to 10–15 larvae per plant specimen (*Carduus hamulosus*), but only 1–3 (maximum five) pupae and adults in the summer. There were dead larvae of 1st and 2nd instars found in one-third of dissected plants. The causes of their deaths are unknown (Figure 6I).

The first pupae were found at the beginning of July (5 July 1983). A young pupa is yellowish-orange (Figure 6J–K). The last pupae were recorded up to the end of July. In their chambers, pupae and adults were typically situated head up and were rarely situated horizontally. Only once was a newly emerged adult in the pupal chamber located head down (Figure 6L). A maximum of six and eight (*Carduus hamulosus* and *Cirsium ukranicum*, respectively) adults successfully finished their development in the same root. According to laboratory observations, the duration of the pupa stage is 14–21 days [83].

**Figure 7:**
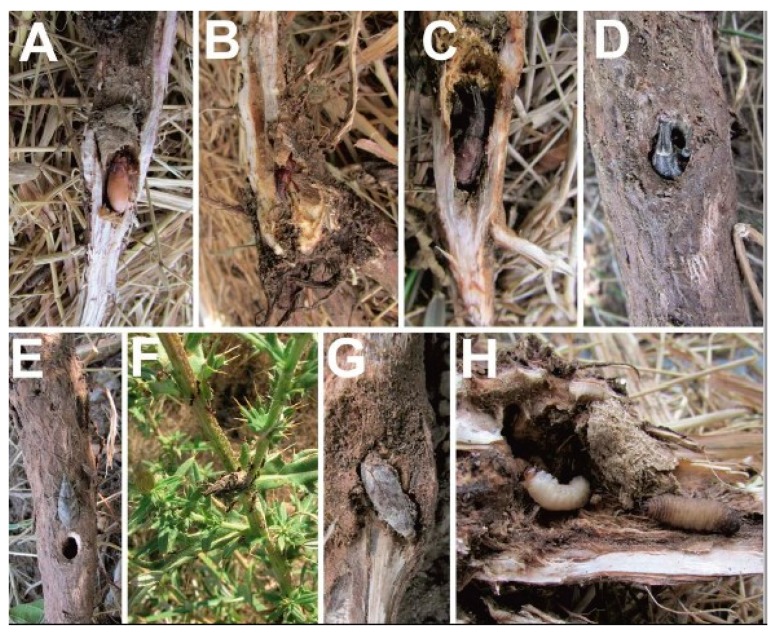
*Cleonis pigra* life history and biotic interactions. (**A**)—The newly emerged adult has light-colored, soft integuments; (**B**)—later, integuments become more solid and reddish-brown; (**C**)—finally, the adult takes on its normal grey color; (**D**)—the emergence of the adult from the stem of *Cirsium ukranicum.* Time: 12:19; (**E**)—the emergence of the adult from the stem of *Cirsium ukranicum.* Time: 12:53; (**F**)—ants (*Lasius*) attack the weevil on *Cirsium ukranicum*; (**G**)—the body of a dead beetle infected by fungi; (**H**)—the larva of *C. pigra* and the larva of a fly (*Cheilosya*) in a root gall on *Cirsium ukranicum*. All photos: S. Volovnik.

After emergence, adults remained in the gall until fully sclerotized, with an upper surface that was usually covered with a pollen-like brownish flush (Figure 7A–C). The adults chewed their way out of the pupal chamber within the root cortex. First, the adult gnawed out a small roundish “window” in the wall. Later, it gnawed small pieces of root cortex near the apex of its rostrum. The adult put the tibia and tarsus of its foreleg into the hole as it became horizontally elongated. This position provided additional support for gnawing. The beetle clenched the wood fibers with its mouthparts, tilted its head right and left, and ripped the fiber. Gnawing went on nonstop; the width of the gnawed area was increased, and the hole enlarged. After 30–40 min had elapsed, the weevils were leaving the gall (Figure 7D–E). Adults of the new generation emerged from the soil and fed on the rosettes of young *Cirsium.* At that stage, the adult can eat up to 120 mg of the leaf tissue at once (60% per primary weight of the body).

Imagines hibernated outside of host plants, most likely in the top layer of soil. Lindenberg [69] wrote that larvae sometimes go from the root to the stem of the Scotch thistle (*Onopordum*) where they finish their development. We never recorded such cases. Hence, *C. pigra* is a univoltine species in Ukraine, and across Europe and North America. There is only one Communication on the life history of *C. pigra* near the eastern limits of its area [86], where the life cycle is different. According to these authors, in Heilongjiang province (northeastern China), newly emerged adults occur in late August and lay eggs in early September. Larvae of the second generation pupate, and the pupae pass the winter outside of the host plants, near its roots. Adults of the next generation occur in mid-May.

#### 3.2.5. Biotic Interactions

In the middle of May 1982, S. Volovnik found many dry fragments of *C. pigra* (mostly bodies without abdomens) on the coastal sands near Popivka in western Crimea. Apparently, these beetles were pecked by water birds. There were two pellets of gulls or other Laridae (Aves, Charadriiformes). The pellets consisted of remnants of 10 and 17 specimens of *C. pigra.* Sometimes (usually in spring), the adults of *C. pigra* are rather numerous. In open habitats (coasts, steppes, and semideserts), these relatively large beetles become the prey of carnivorous animals. In the stomach of steppe birds, namely, the great bustard (*Otis tarda* Linnaeus, 1758) and the little bustard (*Tetrax tetrax* (Linnaeus, 1758) (Aves, Otididae)), as many as nine and eight specimens of *C. pigra*, respectively, were recorded [87]. In other seasons and other habitats, beetles of *C. pigra* are only the accidental prey of many terrestrial vertebrates, especially birds, (e.g., [88,89]), and crabronid wasps from the genus *Cerceris* L. [90,91].

In cavities of the roots of *Centaurea*, the ant *Lasius alienus* (Förster, 1850) (Formicidae, Hymenoptera) and immature stages of *C. pigra* lived divided only by a thin wall, but the ants did not try to get their potential victims [92]. Occasionally, the roots of *Cirsium ukranicum* were surrounded by the nest of *Lasius* sp. This had no effect on the weevils, but ants attacked the weevils upon discovering them on the stems and foliage of the same plant specimen (Figure 7F). Empty galls of *Centaurea diffusa* and *Cirsium ukranicum* became habitats for *Lasius* ants.

Rarely, the pupae and adults of *C. pigra* were infected and destroyed by parasitic fungi [92] (Figure 7G), but it is unknown how this infection occurred there—before or after the insect’s death. In laboratory experiments, a high air humidity results in the appearance of the fungus *Beauveria bassiana* (Bals.-Criv) Vull. on the body of the dead weevils *C. pigra* [93].

Sometimes, after dissecting the root galls of *C. pigra*, we found the cocoons of some parasitoid Hymenoptera (not identified). They passed the winter in the roots they parasitized. In northern Ukraine, up to 80% of *C. pigra* larva were eliminated by the parasitoid wasp *Bracon discoideus* Wesmael, 1838 (Hymenoptera: Braconidae) [69]. From larvae found in southeastern Scotland, seven specimens of the braconid wasp *Bracon sphaerocephalus* Szépligeti, 1901 [94] were reared. In the same region, 20%–60% of *Cleonis* galls were inhabited by the koinobiont endoparasitoid *Acaenitus dubitator* (Panzer, 1800) (Hymenoptera: Ichneumonidae) [95]. The parasitic wasps *Vipio tentator* Rossi, 1790 (Braconidae) and *Aritranis fuscicornis* Tschek, 1871 (Ichneumonidae) attacked late instar larvae in southern Europe [85]. Cawthra [25] also recorded indefinite Diptera and Nematodes as parasites on *C. pigra* larvae.

One author (S.V.) found larval *Cheilosia* sp. (Diptera: Syrphidae) and larval *C. pigra* in the same root tunnel (Figure 7H). Unfortunately, no progress was made to obtain an adult from the larval Diptera. Many of the *Cheilosia* spp. are known mainly as phytophagous, and its larvae often feed on plant tissues in the stems of Asteraceae [96,97,98]. Thus, *Cheilosia* can be a competitor for the larvae of *C. pigra.* Weevils are also infected by saprophagous gnats (*Bradysia* spp., Diptera, Sciaridae [84]).

At times, the larvae of *Cleonis pigra* may coexist peacefully in a compound gall (see above) with the larvae of other gall-making insects that feed on anatomically different parts of the gall; namely, the root collar and root cortex [85]. In general, 14 species of phytophagous insects competed with *C. pigra* in the roots of *Centaurea diffusa* in southern and central Europe [84,99]. These species are separated in time and space. Müller [85] provided an example of such separation: he never found two niche competitors, *C. pigra* and *Cyphocleonus achates* at the same site. Our data differed greatly from that. Those two weevil species were in the same locality, which was the second plot where observations were performed (see Materials and Methods). Both species developed in the roots of *C. diffusa.* Sometimes, the plants infected by one of them were located within several meters of the plant infected with another. Thus, these species could coexist. Because *C. achates* is one of the main biological control agents in North America [100], it should be considered as a biological control agent elsewhere. Only one mature larva (*C. pigra* or *C. achates*, but not both) was recorded per spotted knapweed root. Rarely can two or three larvae be found in the same root [85]. Apparently, the space inside the roots of *Centaurea diffusa* is too limited to support the isolation of multiple larvae, and the larger larva can destroy the smaller larva. Perhaps our second plot (1) was abundant in food and/or (2) one or both weevil species newly settled there. Regardless, neither species was eliminated by direct competition or another ecological mechanism.

In July, Cawthra [25] did not find *C. pigra* larvae in flowering host plants but did find them in non-flowering plants. This could mean that females prefer younger plants for egg laying. This is likely because surface layers of young plants are softer to gnaw for females or newly hatched larvae. Thus, plants that began to grow earlier (before mating and egg laying) were more likely to avoid damage. As a result of larval development, the aboveground part of infested plants becomes significantly smaller and has fewer flowers, but the number of shoots increases [84,99]. The larval development, and probably, gall formation, both cause damage to the transport tissue (xylem). This results in the wilting of thistle (*Cirsium arvense*) and some reduction in the number of flowers. Nevertheless, xylem regenerates and attacked plants rarely die [77]. In spring, when host plants are still young, the damage may result in plant death.

#### 3.2.6. What Are the Other Effects of the Weevil?

Infested specimens of *Silybum marianum* tended to develop additional lateral roots in the soil surface to better absorb atmospheric water [32]. Because adventitious root buds formed on the taproot [101], damage caused by the larval activity of *C. pigra* and gall formation negatively affects the vegetative propagation of *Cirsium*. Along with the flower feeders (e.g., *Larinus* spp.), this can have the synergetic effect of biological control. In general, the activity of both *C. pigra* and *Cyphocleonus achates* has a minor impact on the number of *Centaurea diffusa* plants. There are plots where grass cover is practically absent, but annually, this plant is abundant. If the stem of *Cirsium arvense* breaks down (e.g., by the wind), the break is located above the root crown, and therefore, insects are safe there. The senesced adult plant *C. diffusa* can function as a tumbleweed. A damaged root crown breaks off by wind more easily than a healthy root crown. Therefore, beetle activity indirectly contributes to the dispersal of seeds of its host plant.

## 4. Conclusions

The morphological features of immature *Cleonis pigra* (in both larval and pupal stages) are typical for the subfamily Lixinae. Some original larval morphological features (e.g., the presence of endocarina, the count and position of setae on the labrum, and the count of setae on mandibular) seem to be important features for generic study of the Cleonini. Keys for the determination of selected Cleonini species based on their larval and pupal characteristics are attached. Additionally, Dyar’s law was also used to estimate a number of larval instars of *C. pigra.* Moreover, the description of habitats, adult behavior, host plants, life cycle, and biotic interactions are discussed in detail here. Finally, it seems that such studies should improve the current knowledge of the weevil distributions and should provide practical guidance in the use of this group as a potential biological control agent.

## Figures and Tables

**Table 1:** Key to the Known Larvae of Cleonini.

**1.**	Endrocarina present.	**2**
**-**	Endrocarina absent.	**9**
**2.**	Labrum with three *lrms.*	**3**
**-**	Labrum with two *lrms.*	***Scaphomorphus erysimi***
**3.**	Setae on labrum (*lrms*) in a triangle position.	**4**
**-**	Setae on labrum (*lrms*) in a line.	**6**
**4.**	Dorsum on abdominal segments I–VII with six or more *pds*.	
	Ligula with three setae. Mandibula with two setae.	**5**
**-**	Dorsum on abdominal segments I–VII with four *pds*. Ligula with two setae. Mandibula with one seta.	***Asproparthenis carinicollis***
**5.**	Dorsum on abdominal segments I–VII with six *pds* (not in one line) and two *ss* (one not in line with *pds*). Dorsum on abdominal segment VIII with five *pds* and two *ss.*	***Adosomus roridus***
**-**	Dorsum on abdominal segments I–VII with seven *pds* and one *ss* (all in one line). Dorsum on abdominal segment VIII with five *pds* and one *ss.*	***Cleonis pigra***
**6.**	Head with four *des.*	**7**
**-**	Head with five *des.*	**8**
**7.**	Two *des* more than twice as long as the remaining two setae	***Bothynoderes declivis***
**-**	All four *des* of almost the same size	***Bothynoderes affinis***
**8.**	Mandibula with one seta. Maxilla with one *mbs.*	***Cyphocleonus achates***
**-**	Mandibula with two setae. Maxilla without *mbs.*	***Rhabdorrhynchus karelinii***
**9.**	Setae on labrum (*lrms*) in a triangle position.	**10**
**-**	Setae on labrum (*lrms*) in a line.	***Pachycerus scabrosus***
		**sensu Scherf (1964)**
**10.**	Mandibula with two setae. Epipharynx with four *als*. Ligula with two setae.	***Cyphocleonus dealbatus***
**-**	Mandibula with one seta. Epipharynx with six *als*. Ligula with three setae.	***Coniocleonus nigrosuturatus***

**Table 2:** Key to the Known Pupae of Cleonini.

**1**	Mesonotum with five or six setae. Dorsum of abdominal segments I–VI with nine or ten setae (sometimes setae replaced by thorn-like asperities).	**2**
**-**	Mesonotum with three setae. Dorsum of abdominal segments I–VI with six or eight setae (sometimes setae replaced by thorn-like asperities).	**6**
**2.**	Head with two or three *sos.*	**3**
**-**	Head with one *sos*. Mesonotum with five setae. Dorsum of abdominal segments I–VI with ten setae.	***Rhabdorrhynchus karelinii***
**3**	Mesonotum with six setae.	**4**
**-.**	Mesonotum with five setae. Rostrum with four *rs*. Dorsum of abdominal segments I–VI with nine setae. Each apex of femora with two *fes.*	***Cleonis pigra***
**4**	Rostrum with one *rs*. Dorsum of abdominal segments I–VI with nine setae.	**5**
**-**	Rostrum with two *rs*. Dorsum of abdominal segments I–VI with ten setae. Each apex of femora with two *fes.*	***Adosomus roridus***
**5.**	Pronotum with ten setae. Head with four *pas*. Each apex of femora with three *fes.*	***Coniocleonus nigrosuturatus***
**-**	Pronotum with eleven setae. Head with five *pas.*	***Scaphomorphus*** ***erysimi***
**6**	Head with two *sos*. Metanotum with three setae.	**7**
**-.**	Head with one *sos*. Metanotum with four setae.	***Bothynoderes affinis***
**7.**	Dorsum of abdominal segments I–VI with six setae.	***Asproparthenis carinicollis***
**-**	Dorsum of abdominal segments I–VI with eight setae.	***Cyphocleonus achates***
